# Metabolic Characteristics and Cytokine Gene Polymorphisms as Potential Risk Factors for a Higher Liver Fibrosis Stage in MASLD Patients: A Hospital-Based Study

**DOI:** 10.3390/ijms26083730

**Published:** 2025-04-15

**Authors:** Mihaela Iancu, Sorina-Cezara Coste, Angela Cozma, Olga Hilda Orășan, Roxana Liana Lucaciu, Adriana Corina Hangan, Ioana Para, Sidonia Gog Bogdan, Lucia-Maria Procopciuc

**Affiliations:** 1 Medical Informatics and Biostatistics, Faculty of Nursing and Health Science, “Iuliu Hațieganu” University of Medicine and Pharmacy, 400012 Cluj-Napoca, Romania; miancu@umfcluj.ro; 2 4th Department of Internal Medicine, Faculty of Medicine, “Iuliu Hațieganu” University of Medicine and Pharmacy, 400012 Cluj-Napoca, Romania; angelacozma@umfcluj.ro (A.C.); hilda.orasan@umfcluj.ro (O.H.O.); ioana.para@yahoo.com (I.P.); 3 Department of Pharmaceutical Biochemistry and Clinical Laboratory, Faculty of Pharmacy, “Iuliu-Hațieganu” University of Medicine and Pharmacy, 400012 Cluj-Napoca, Romania; liana.lucaciu@umfcluj.ro; 4 Department of Inorganic Chemistry, Faculty of Pharmacy, “Iuliu-Hațieganu” University of Medicine and Pharmacy, 400012 Cluj-Napoca, Romania; adriana.hangan@umfcluj.ro; 5 Department of Surgery and ATI, Faculty of Veterinary Medicine, University of Agricultural Sciences and Veterinary Medicine, 400372 Cluj-Napoca, Romania; sidonia.bogdan@usamvcluj.ro; 6 Medical Biochemistry, Department of Molecular Sciences, Faculty of Medicine, “Iuliu Hațieganu” University of Medicine and Pharmacy, 400349 Cluj-Napoca, Romania; lprocopciuc@umfcluj.ro

**Keywords:** MASLD, liver fibrosis, interleukin 17, toll-like receptor 4, gene polymorphisms

## Abstract

Polymorphisms in the Toll-like receptor 4 (TLR4) and IL-17 cytokine genes play a role in liver fibrosis progression among patients with MASLD. The current study aimed to investigate whether the *IL17* (*A7448G* and *G197A*) and *TLR4* (*Asp299Gly* and *Thr399Ile*) gene polymorphisms are associated with increased liver fibrosis stages in MASLD patients. Genotyping for the *IL17F*-*A7488G*, *IL17A*-*G197A*, *TLR4*-*Asp299Gly*, and *TLR4*-*Thr399Ile* polymorphisms was performed on a sample of 42 MASLD patients and 39 healthy controls. Serum levels of IL17F, IL17A, and TLR4 were measured using ELISA techniques. Bivariate analysis revealed significant associations between glycemic levels (*p* = 0.006), lipid metabolism (total cholesterol, HDL cholesterol, triglycerides), and the severity of liver fibrosis (*p* < 0.05). The *IL17A*-*G197A* GA and AA genotypes were more frequent in patients with advanced liver fibrosis compared to those without fibrosis (GA genotype frequency: 42.9% vs. 7.7%; AA genotype frequency: 14.3% vs. 5.1%; adjusted *p* = 0.0423). In the multivariable ordinal logistic regression, the *IL17A*-*G197A* polymorphism remained significantly associated with higher liver fibrosis stages (adjusted *p* = 0.0155). Patients with the dominant genotype (GA + AA) of the *IL17A*-*G197A* polymorphism exhibited 3.91 times greater odds of experiencing at least a one-stage increase in liver fibrosis compared to those with the GG genotype (adjusted OR = 3.91, 95% CI: 1.33–12.34). This study indicates that *IL17*-related genetic polymorphisms and metabolic characteristics significantly affect liver fibrosis progression in MASLD patients, with the *IL17A*-*G197A* gene polymorphism identified as an independent multivariate predictor of fibrosis progression.

## 1. Introduction

Metabolic dysfunction-associated steatotic liver disease (MASLD), as the name suggests, is characterized by the accumulation of fat in the liver, primarily caused by metabolic disorders. Previously known as non-alcoholic fatty liver disease (NAFLD), MASLD encompasses a broad spectrum of liver conditions, ranging from simple steatosis (MAFL) to more severe forms such as non-alcoholic steatohepatitis (MASH) and, in certain cases, progression to liver fibrosis or even cirrhosis (MASH cirrhosis) [1]. Liver fibrosis results from the excessive accumulation of extracellular matrix proteins, which can lead to scarring and impaired liver function [2].

In MASLD, persistent inflammation and hepatocellular damage can trigger the fibrogenesis process, which promotes the development of fibrosis. This issue is particularly concerning, because liver fibrosis is a significant predictor of morbidity and mortality in patients with liver disease [3]. Diagnosing MASLD typically involves a clinical evaluation of patients with two or more metabolic risk factors (e.g., obesity, dyslipidemia, established or early-stage type 2 diabetes, hypertension) or patients who have first-degree relatives with MASLD-associated cirrhosis. Investigations include blood tests to assess liver enzymes and imaging studies, such as ultrasound and magnetic resonance imaging (MRI) [4]. However, liver biopsy remains the gold standard for evaluating fibrosis, although non-invasive methods like FibroScan and specific serum biomarkers are becoming increasingly prevalent [5]. MASLD is currently a major public health issue globally, affecting millions of people and placing considerable strain on healthcare systems worldwide [6]. It is closely linked to numerous metabolic disorders and may lead to severe complications like cirrhosis and hepatocellular carcinoma [7]. Therefore, early diagnosis and targeted therapeutic interventions are vital, along with the implementation of public health strategies to reduce the effects of MASLD. Addressing the rise in MASLD cases is critical for managing the worldwide epidemic of metabolic and liver diseases [8].

In addition to the well-known risk factors of MASLD, including obesity, hypertension, dyslipidemia, and type 2 diabetes, genetic factors also play a significant role in the pathogenesis of this condition [1]. Polymorphisms in the Toll-like receptor 4 (*TLR4*) and *IL-17* cytokine genes are involved in the progression of liver fibrosis in patients with MASLD. Studies indicate that single-nucleotide polymorphisms (SNPs) of TLR4, such as *D299G* and *T399I*, have a protective effect against fibrosis progression, because they reduce TLR4-mediated inflammatory signaling in hepatic stellate cells, thereby decreasing the risk of fibrosis [9]. Moreover, recent research shows that fibrosis progression in MASLD is influenced by gut microbiota dysbiosis and genetic variants, such as PNPLA3, which, along with the *TLR4* and *IL17* SNPs, contribute to the advancement of fibrogenesis [10]. These findings suggest that polymorphisms of the *TLR4* and *IL17* genes, together with changes in the gut microbiota, play a central role in the development and progression of liver fibrosis.

Furthermore, genetic testing for *TLR4* and *IL17* polymorphisms may help predict the progression of MASLD by identifying individuals with a higher or lower risk of liver fibrosis. TLR4 polymorphisms, such as *Asp299Gly* and *Thr399Ile*, have been shown to modulate inflammatory responses; thus, carriers of these variants may experience a slower fibrosis progression due to reduced inflammatory signaling. Similarly, polymorphisms of the *IL17* gene, particularly those affecting *IL-17A* and *IL-17F*, can influence the production of IL-17, a cytokine vital for mediating inflammation and liver fibrosis. Although *TLR4* SNPs, such as *rs4986790*, have shown protective effects in other conditions, such as viral infections [11], emerging evidence suggests that these mechanisms may also impact the course of liver disease in MASLD. However, further studies are required to establish predictive models based on these polymorphisms in the context of MASLD [12].

Cytokine IL-17 plays a crucial role in the progression of liver fibrosis, and its elevated serum levels correlate with the severity of the disease. In cases of liver fibrosis, particularly concerning chronic conditions such as viral hepatitis B and C, IL-17 contributes to amplifying the inflammatory response and stimulating the fibrogenesis process. Studies have demonstrated that patients with liver fibrosis and cirrhosis exhibit increased levels of IL-17, which positively correlate with advanced stages of liver disease, including the development of hepatocellular carcinoma [13]. IL-17 exacerbates liver damage by intensifying the profibrotic effects of other cytokines, such as TGF-β1 and IL-13, which provoke a pronounced fibrotic response in the liver, as shown in the study by Abd Allah et al. (2022) using animal models of Schistosoma mansoni infection [14]. Thus, IL-17 serves as both a marker and a mediator of liver fibrosis, highlighting its significant potential as a therapeutic target to slow the progression of fibrosis in chronic liver diseases.

Elevated serum levels of Toll-like receptor 4 (TLR4) have been shown to play a significant role in the progression of liver fibrosis, particularly in nonalcoholic steatohepatitis (NASH) and other chronic liver diseases, such as viral hepatitis C. Studies indicate that serum TLR4 levels are directly correlated with the degree of liver fibrosis in patients with NASH, with a progressive increase in these levels observed as fibrosis advances from the F0 to F4 stage [15]. TLR4 is crucial for activating proinflammatory pathways in the liver, and its expression has been linked to portal inflammation and the activation of fibrogenic cells, significantly contributing to the progression of fibrosis [16]. Additionally, in patients with chronic viral hepatitis C, TLR4 expression correlates with advanced stages of fibrosis [17], underscoring its role as a key mediator of inflammation and fibrogenesis in liver disease. These findings suggest that TLR4 could serve as a biomarker for assessing the severity of fibrosis and might also be a potential therapeutic target for slowing fibrosis progression.

The objectives of this study were the following: (i) to explore whether the *IL17* (*A7448G* and *G197A*) and *TLR4* (*Asp299Gly* and *Thr399Ile*) gene polymorphisms act as risk factors for advanced liver fibrosis stages in MASLD patients; (ii) to examine the association between plasma levels of *IL17A/F* and *TLR4,* and *IL17* (*A7448G* and *G197A*) and *TLR4* (*Asp299Gly* and *Thr399Ile*) gene polymorphisms, stratified by liver fibrosis stages.

## 2. Results

### 2.1. Description of the Studied Groups

The demographics and clinical characteristics of the MASLD patients and controls are summarized in Table 1. Comparative analysis indicated no significant differences in mean age, sex distribution, smoking rates, hypertension prevalence, mean LDL cholesterol, SBP levels, AST levels, total bilirubin levels, and INR levels among the three studied groups (Table 1). However, the mean BMI levels significantly differed between the groups (*p* = 0.00005), with higher values noted in the group with advanced fibrosis. Post hoc analysis revealed significant differences only between controls and patients with mild to moderate fibrosis (Games–Howell test, adjusted *p* = 0.00015) and between controls and patients with advanced fibrosis (Games–Howell test, adjusted *p* = 0.0004). Additionally, significant differences were found in the mean waist circumference between liver fibrosis stages (Table 1), with significant differences observed only between controls and patients with mild to moderate liver fibrosis (Tukey test, adjusted *p* = 0.001).

According to Kruskal–Wallis’ test, serum levels of alkaline phosphatase and GGT differed significantly among the studied groups, and a post hoc analysis conducted using Dunn’s test revealed elevated levels of these liver parameters in patients with mild to moderate fibrosis and the advanced fibrosis group compared to controls (Table 1)

### 2.2. Association of Metabolic Factors and Gene Polymorphisms with Liver Fibrosis Severity

Bivariate analysis revealed significant associations between glycemic values (*p* = 0.006), lipid metabolism characteristics (total cholesterol, HDL cholesterol, triglycerides), and liver fibrosis severity (Table 2). Post hoc analysis revealed that the levels of metabolic features differed significantly between patients with no liver fibrosis and those with advanced fibrosis, indicating an increase in metabolic parameters as liver fibrosis progresses (Table 2). The *IL17A-G197A* GA and AA genotypes were more frequent in patients with advanced liver fibrosis compared to those with no fibrosis (GA genotype frequency: 42.9% vs. 7.7%; AA genotype frequency: 14.3% vs. 5.1%; adjusted *p* = 0.0423) and were also more prevalent in patients with mild to moderate liver fibrosis compared to those with no fibrosis (GA genotype frequency: 40.0% vs. 7.7%; AA genotype frequency: 11.4% vs. 5.1%; adjusted *p* = 0.0014). Significant differences were also noted for the G allele of the *IL17F-A7448G* (adjusted *p* = 0.0003) and the A allele of the *IL17A-G197A* gene polymorphisms (adjusted *p* = 0.0338), which were more common in patients with advanced liver fibrosis than in those with no fibrosis (Table 2).

### 2.3. Effects of the IL17 (A7488G, G197A) and TLR4 (Asp299Gly, Thr399Ile) Gene Polymorphisms on the Risk of Having at Least a One-Stage Increase in Liver Fibrosis Among MASLD Patients

A higher stage of liver fibrosis was significantly associated with the dominant genotype of the *IL17A-G197A* gene polymorphism (*p* = 0.0004). The proportion of patients with mild to moderate fibrosis increased from 31.5% in those with the homozygous wild GG genotype of the *IL17A-G197A* gene polymorphism to 66.7% in patients carrying the GA + AA variant genotype. In contrast, the proportion of advanced fibrosis increased from 5.6% to 14.8%.

### 2.4. Effects of Metabolic Factors on the Risk of Having at Least a One-Stage Increase in Liver Fibrosis Among MASLD Patients

Concerning metabolic factors, fasting blood glucose, total cholesterol, HDL cholesterol, LDL cholesterol, and triglycerides emerged as significant univariate predictors for higher liver fibrosis stages in univariable logistic models (Table 3). Each unit increase in fasting blood glucose, total cholesterol, LDL cholesterol, and triglycerides was significantly associated with a higher risk of experiencing at least a one-stage increase in liver fibrosis (Table 3).

### 2.5. Combined Effects of Genetic and Metabolic Factors on the Risk of Having at Least a One-Stage Increase in Liver Fibrosis

In the multivariable ordinal logistic model, only the *IL17A-G197A* gene polymorphism was significantly associated with a higher liver fibrosis stage (adjusted *p* = 0.0155); none of the other metabolic or genetic factors demonstrated a significant association with an increased liver fibrosis stage (Table 3). Patients with the dominant genotype (GA + AA) of the *IL17A-G197A* gene polymorphism had 3.91-fold higher odds of experiencing at least a one-stage increase in liver fibrosis compared to those with the GG genotype (adjusted OR = 3.91, 95% CI: 1.33–12.34).

### 2.6. Associations Between Plasma Levels of IL17 Cytokines, TLR4, IL17 (A7488G, G197A), and TLR 4 (Asp299Gly, Thr399Ile) Gene Polymorphisms Stratified by Liver Fibrosis Stages

**Figure 1:**
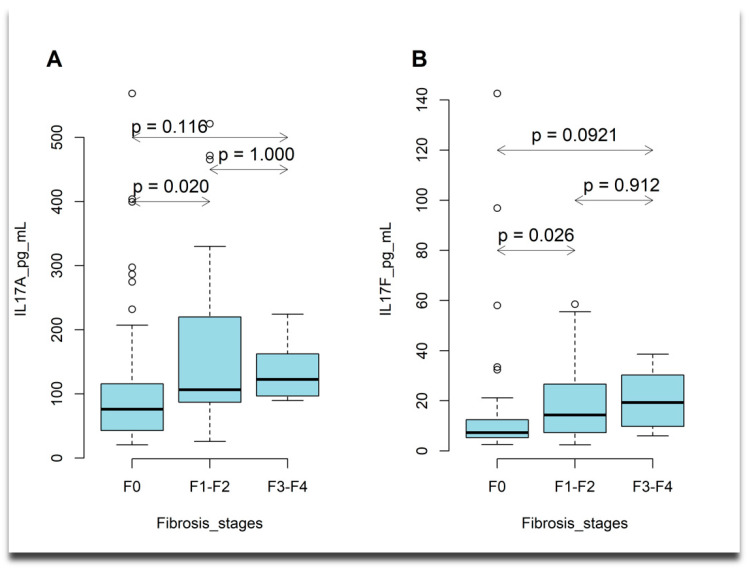
(**A**) Box plots describing plasma levels of IL17A cytokine increases with liver fibrosis stage. Horizontal line inside the box shows the median level of the studied cytokine, the boxes denote the interquartile range [Q1, Q3], and outliers are indicated by dots; estimated *p*-values are adjusted *p*-values obtained from Dunn’s test with a Bonferroni correction. (**B**) Box plots describing plasma levels of IL17F cytokine increases with liver fibrosis stage. Horizontal line inside the box shows the median level of the studied cytokine, the boxes denote the interquartile range [Q1, Q3], where Q1 = first quartile, Q3 = third quartile, and outliers are indicated by dots; estimated *p*-values are adjusted *p*-values obtained from Dunn’s test with a Bonferroni correction.

**Figure 2:**
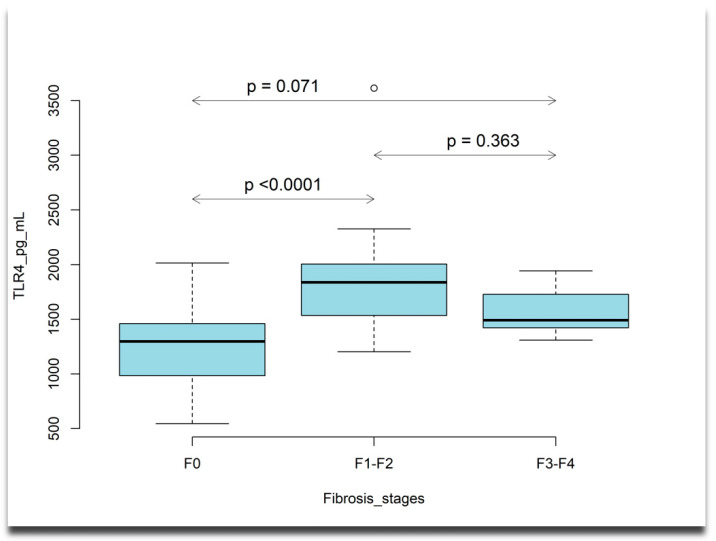
Box plots describing plasma levels of TLR4 increases with the liver fibrosis stage. Horizontal line inside the box shows the median level of the studied cytokine, the boxes denote the interquartile range [Q1, Q3] where Q1 = first quartile, Q3 = third quartile, and outliers are indicated by dots; estimated *p*-values are adjusted *p*-values obtained from Dunn’s test with a Bonferroni correction.

The plasma levels of the IL17A cytokine (Kruskal–Wallis test, *p* = 0.02427), IL17F cytokine (Kruskal–Wallis test, *p* = 0.02575), and TLR4 (Kruskal–Wallis test, *p* < 0.0001) were significantly different among patients with various stages of liver fibrosis. Overall, we observed an increase in the plasma levels of IL17 cytokines as the fibrosis stage progressed, but this increase was not statistically significant with each stepwise increase in the liver fibrosis grade (Figure 1). The plasma level of TLR4 differed across liver fibrosis stages, with patients in advanced fibrosis stages showing lower levels of TLR4 compared to those with moderate fibrosis (Figure 2).

As shown in Table 4, no significant differences in plasma levels of IL17 cytokines and TLR4 were found between MASLD patients carrying the dominant variant genotypes of the IL17 (A7488G, G197A) and TLR 4 (Asp299Gly, Thr399Ile) gene polymorphisms and those with the wild-type genotype (*p* > 0.05).

## 3. Discussion

Identifying new methods for assessing liver fibrosis in MASLD, formerly known as NAFLD, is becoming increasingly important, given the close link between this condition and the risk of progression to cirrhosis, with liver failure or hepatocarcinoma. Liver fibrosis is a critical stage in the evolution of MASLD, and early diagnosis is essential to prevent severe complications. Various recent studies have highlighted the importance of an accurate fibrosis risk assessment and proposed new tools for this purpose.

A recent study by Chen et al. (2024) compared several blood-based fibrosis scores, such as LiverRisk and SAFE, in different populations, including in patients with MASLD. Although these scores showed good performance in the general population, their accuracy in patients with MASLD varies significantly, especially in those with comorbidities such as type 2 diabetes [18]. This variation underlines the need to develop tools that are better adapted to the complexity of the metabolic disease associated with MASLD. In addition, the study by Huang et al. (2024) investigated the effect of moderate alcohol consumption on liver fibrosis in patients with MASLD, suggesting a possible protective effect. However, the protection offered by moderate alcohol consumption decreases significantly in patients with type 2 diabetes, which indicates that therapeutic recommendations should be personalized according to the metabolic profile of each patient [19]. For pediatric MASLD, new risk scores, such as pFIB-c, have recently been validated and have proven to be effective in excluding significant fibrosis, with a good performance in non-tertiary settings. This development is promising for improving early diagnosis in children and adolescents [20].

In terms of non-invasive investigations, a new score, the LSS (Liver Stiffness Score), has shown promise in detecting advanced fibrosis among patients with MASLD, demonstrating superior performance compared to existing scores such as FIB-4 [21]. These findings underscore the need to develop and refine fibrosis assessment methods to ensure a more accurate diagnoses and early therapeutic interventions in MASLD.

In conclusion, progress in the discovery of more accurate and specific diagnostic methods for liver fibrosis in the context of MASLD is crucial for improving patients’ prognoses and preventing serious complications such as cirrhosis and hepatocarcinoma.

Genetic polymorphisms play a central role in the progression of liver fibrosis in patients with MASLD, significantly influencing the inflammatory and apoptotic mechanisms involved in fibrogenesis. In particular, *TLR4* polymorphisms, such as *D299G* and *T399I*, have a major impact on the evolution of fibrosis by modulating the activation of TLR in hepatic stellate cells (HSCs). These genetic variants reduce the activation of the TLR4-mediated NF-κB pathway, leading to a decreased production of proinflammatory cytokines, such as IL-6 and MCP-1, and an increased apoptosis rate of HSCs. The reduced activity of NF-κB decreases the levels of Bcl-2, an anti-apoptotic protein, facilitating the elimination of activated liver stellate cells, key elements in the progression of liver fibrosis. Thus, *TLR4* polymorphisms, such as *D299G* and *T399I*, reduce the risk of fibrosis by weakening fibrogenic pathways and amplifying the susceptibility of HSCs to apoptosis [9].

Also, polymorphisms of the *IL-17* gene play a significant role in the evolution of liver fibrosis in patients with MASLD. *IL17* polymorphisms, such as *rs8193036* and *rs2275913*, have been studied for their role in chronic liver diseases, such as hepatitis, but their direct association with fibrosis in MASLD requires further investigation. Although these variants do not have a strong and consistent association with susceptibility to liver fibrosis progression in all populations, they emphasize the critical role of IL-17 in perpetuating chronic inflammation and fibrogenesis in MASLD. IL-17, produced mainly by Th17 cells, has a major proinflammatory impact by recruiting immune cells and stimulating liver fibrogenesis [22]. High serum levels of IL-17 have been correlated with fibrosis severity and progression to more advanced stages such as cirrhosis and hepatocellular carcinoma [23,24].

The current research highlights crucial connections among metabolic factors, genetic variations, and the extent of liver fibrosis in MASLD patients. Notably, significant increases in BMI and waist circumference were observed in those with liver fibrosis, especially among patients with advanced fibrosis. Moreover, significant bivariate associations were observed between hyperglycemia, dyslipidemia, and the severity of fibrosis. In addition, regression analysis revealed that each unit increase in fasting blood glucose, total cholesterol, LDL cholesterol, and triglycerides was significantly associated with a higher likelihood of experiencing at least a one-stage progression in liver fibrosis. These findings align with the research by Bansal et al., which underscored how dyslipidemia and hyperglycemia can accelerate fibrosis progression through a heightened lipotoxicity and hepatic insulin resistance [25]. The observed alterations in lipid metabolism may reflect hepatic insulin resistance and increased lipotoxicity, both of which play a role in fibrogenesis.

An analysis of genetic data revealed that the *IL17A-G197A* GA and AA genotypes were more prevalent in patients with advanced liver fibrosis than in those without fibrosis (GA genotype frequency: 42.9% vs. 7.7%, AA genotype frequency: 14.3% vs. 5.1%, adjusted *p* = 0.0423). Similarly, these genotypes were found more frequently in patients with mild to moderate liver fibrosis, compared to those with no fibrosis (GA genotype frequency: 40.0% vs. 7.7%, AA genotype frequency: 11.4% vs. 5.1%, adjusted *p* = 0.0014). IL17A plays a significant role in hepatic inflammation by increasing the production of pro-fibrotic cytokines and activating hepatic stellate cells. Thielemann et al. noted a related association, identifying the *IL17A rs2275913* minor allele variant as a risk factor for fibrosis progression in MASLD patients, highlighting its significance in inflammation and immune dysregulation [26]. Additionally, the present findings regarding the *IL17F-A7448G* and *TLR4* polymorphisms in advanced fibrosis corroborate the results reported by Guo et al., who demonstrated that these variations contribute to immune dysregulation and chronic liver inflammation, thereby emphasizing their role in hepatic fibrogenesis [9].

The multivariable analysis conducted in this study highlights the independent association of the *IL17A-G197A* polymorphism with fibrosis severity, suggesting its potential as a genetic marker for assessing fibrosis progression risk. Patients possessing the dominant genotype (GA + AA) of the *IL17A-G197A* gene polymorphism have a risk 3.91 times greater of developing at least a one-stage increase in liver fibrosis than individuals with the GG genotype (adjusted OR = 3.91, 95% CI: 1.33–12.34). In line with the present findings, recent research indicates a heightened presence of *IL17A* variants in MASLD patients experiencing advanced fibrosis, implying that targeting the IL17 pathway could offer therapeutic benefits [24]. The research of Ge et al. explored the *IL17A-G197A* gene polymorphism and found that *IL17A-G197A* was associated with a significant risk of liver cirrhosis susceptibility (OR = 4.186, *p* = 0.032) in a Chinese HVB-infected cohort, supporting the polymorphism’s role in fibrosis progression in viral liver disease as well [27].

In the current study, plasma concentrations of IL-17A and IL-17F increased in tandem with the extent of fibrosis. This observation aligns with previous studies conducted by Massabayeva et al. [23] and Olveira et al. [24], both of which established a connection between IL-17 levels and the severity of fibrosis. This suggests a role for IL-17 in hepatic inflammation and the progression of the disease.

Findings from this research highlight the link between metabolic and genetic factors in the progression of fibrosis, yet certain limitations must be acknowledged. The limited sample size may have influenced its statistical power, particularly in the advanced fibrosis group. Furthermore, the cross-sectional nature of this study constrains causal interpretations regarding fibrosis progression. Future longitudinal studies involving larger cohorts are essential to validate these findings and explore the underlying mechanisms of these associations.

While current fibrosis risk scores rely on biochemical markers, incorporating genetic polymorphisms such as *IL17A-G197A* and *TLR4* variants into predictive models may enhance risk stratification in MASLD. Future studies should confirm whether these genetic markers improve the accuracy of fibrosis assessment tools like LSS.

The role of IL-17 in fibrosis progression is well established, but its potential as a therapeutic target remains underexplored. Further research should assess whether inhibiting IL-17 can slow fibrosis progression in MASLD patients

## 4. Materials and Methods

### 4.1. Study Design

The present study included a total of 81 subjects who presented for routine analysis at CFR University Clinical Hospital in Cluj-Napoca, Romania, between October 2018 and August 2022. The study design was a case–control study. The case group included 42 patients diagnosed with MASLD, while the control group consisted of 39 subjects without MASLD at the time of hospital admission. This study received approval from the medical ethics commission of the University of Medicine and Pharmacy “Iuliu Hatieganu”, Cluj-Napoca (322/26 July 2018) and was conducted according to the principles outlined in the Declaration of Helsinki. Written informed consent was obtained from all participants prior to their inclusion in the study.

Patients were enrolled based on the presence of metabolic dysfunction, identified by at least one of the following five metabolic criteria: (1) fasting glucose ≥ 100 mg/dL or the use of antidiabetic medications; (2) low HDL cholesterol (<40 mg/dL in men, <50 mg/dL in women, or the use of statin medications); (3) hypertriglyceridemia (TG ≥ 150 mg/dL or the use of lipid-lowering therapy); (4) central obesity (waist circumference ≥ 102 cm in men, ≥88 cm in women); and (5) hypertension (blood pressure ≥ 130/85 mmHg or antihypertensive treatment). Hepatic steatosis was diagnosed through imaging (ultrasound) or histological examination, while alcohol consumption (>30 g/day in men, >20 g/day in women) was a criterion for exclusion [28]. Additional exclusion criteria included liver diseases of other etiologies, such as alcoholic hepatitis, viral hepatitis B and C, Wilson’s disease, autoimmune hepatitis, and hemochromatosis, as well as systemic conditions known to influence IL17 and TLR4 polymorphisms, including asthma, rheumatoid arthritis, and inflammatory bowel disease.

### 4.2. Clinical and Biochemical Measurements

The patient data collected included age, sex, body mass index (BMI), waist circumference, smoking habits, and blood pressure. Fasting blood samples were subjected to biochemical analysis, which evaluated glucose, total cholesterol, HDL cholesterol, triglycerides, liver enzymes (AST, ALT), alkaline phosphatase (ALP), gamma transferase (GGT), platelet count, serum bilirubin, erythrocyte sedimentation rate (ESR), and C-reactive protein (CRP).

### 4.3. Assessment of Hepatic Steatosis and Fibrosis

Hepatic steatosis was diagnosed through ultrasound, using echogenicity comparisons between the liver and kidneys following American Gastroenterological Association guidelines [29]. In selected patients, an ultrasound-guided liver biopsy was performed, followed by histological evaluation.

Two specialized liver pathologists conducted liver biopsy readings for both cohorts, simultaneously. Steatosis was scored according to grades 0–3 [30,31], and fibrosis was assessed on a scale from stage F0 to F4. Stage F0 denotes no fibrosis, stage F1 indicates perisinusoidal or periportal fibrosis, stage F2 reflects perisinusoidal and (peri)portal fibrosis, stage F3 signifies bridging fibrosis, and stage F4 represents cirrhosis.

### 4.4. Serum IL17A/F and TLR4 Measurements

The serum concentrations of IL17A/F and TLR4 were determined using an enzyme-linked immunosorbent assay (ELISA), according to the methodology used in our previous study [32]. Microplates pre-coated with specific antibodies for IL17A/F and TLR4 were used (BioVendor Laboratorni Medicina a.s.; Elabscience Biotechnology Inc., Houston, TX, USA), following the manufacturer’s protocol (IL17A—Catalog No: E-EL-H0105; IL17F—Catalog No: RAF043R; TLR4—Catalog No: E-EL-H6123). The optical density (DO) reading at 450 nm was performed spectrophotometrically using a microplate reader (Absorbance Microplate Reader Sunrise Tecan; Tecan Group Ltd., Männedorf, Switzerland), and the washing of the plates was carried out with Biochrom Asys Atlantis Microplate Washer (Biochrom Ltd., Cambridge, UK). This method allowed the precise quantification of serum levels of IL17A, IL17F, and TLR4, facilitating the analysis of their association with the investigated genetic factors.

### 4.5. Genetic IL17 A/F and TLR4 Polymorphism Analysis

Genomic DNA was extracted from peripheral blood samples (5 mL) collected from patients and controls in EDTA tubes, using a Quick-DNA Miniprep kit (Zymo Research Corporation, Freiburg, Germany). The DNA samples were stored at −20 °C until genotyping analyses were performed, according to the methodology used in our previous study [31]. The genotyping of *IL17F*-*A7488G*, *IL17A*-*G197A*, *TLR4-Asp299Gly* and *TLR4*-*Thr399Ile* polymorphisms was performed by polymerase chain reaction (PCR) and restriction fragment polymorphism (RFLP) analysis. The procedure was based on methods described in the literature and optimized in our laboratory to ensure the reproducibility and accuracy of the results. The genomic DNA was amplified using a Bio-Rad thermo-cycler (Bio-Rad Life Science, Hercules, CA, USA). The composition of the reaction mixture (25 μL) included 5 μL DNA template, 0.2 μM primers, 200 μM dNTPs, PCR buffer, 2.0 mM MgCl_2_, and 0.625 U FIREPol DNA polymerase. The amplicons were digested with restriction enzymes (New England Biolabs, Ipswich, MA, USA) and visualized on 2% agarose gels stained with ethidium bromide under UV transillumination. After PCR amplification, 6 μL of the amplified product was digested with 2U restriction enzyme for 3 h at 37 °C. The digestion products were analyzed by electrophoresis on 3% agarose gel, stained with ethidium bromide and visualized under UV to confirm the specificity of the RFLP method. This methodology allowed the identification of the studied polymorphisms, providing relevant results for the genetic analysis of patients.

### 4.6. Statistical Analysis

In the current study, qualitative characteristics were described using counts and relative frequencies. Differences in the frequency distribution of qualitative characteristics between the studied groups (no fibrosis, mild to moderate fibrosis, and advanced fibrosis) were tested using Fisher’s exact test, followed by pairwise Fisher’s exact test post hoc comparisons.

After testing for a normal distribution (Shapiro–Wilk’s test, quantile–quantile plots, method of moments [33]), the quantitative characteristics were summarized by the mean value with standard deviations or medians with interquartile ranges (lower quartile, Q1, upper quartile, Q3).

The comparison of quantitative characteristics across the studied groups was conducted using a one-way analysis of variance (ANOVA) with Tukey’s Honest Significant Difference (HSD) for post hoc multiple comparisons, or Welch’s ANOVA followed by the Games–Howell test or the Kruskal–Wallis test followed by Dunn’s post hoc test, depending on the distribution of continuous variables within the groups.

To evaluate the associations of metabolic characteristics and *IL17* (*A7448G* and *G197A*) and *TLR4* (*Asp299Gly* and *Thr399Ile*) gene polymorphisms with the risk of a higher fibrosis stage, we tested univariable ordinal logistic models. Explicative variables with a significance level of *p* < 0.10 were subsequently included in a multivariable ordinal logistic model.

The association effect size was quantified using odds ratios (ORs), which represented the risk of at least a one-stage increase (progression) in liver fibrosis severity, without assuming that the magnitude of each one-stage increase was uniform across liver fibrosis categories. The ORs were reported alongside their corresponding 95% confidence intervals.

Statistical significance was assumed at a two-sided estimated level (p), lower than α = 0.05. All statistical analyses were conducted using R software (R Foundation for Statistical Computing, Vienna, Austria), version 4.4.2.

## 5. Conclusions

In summary, evidence from this study indicates that metabolic characteristics and *IL17*-related genetic polymorphisms significantly influence the progression of liver fibrosis in patients with MASLD. Identifying *IL17A-G197A* as a standalone predictor of fibrosis progression underscores its potential value in risk assessment and targeted treatment strategies. Future studies should aim to clarify the specific molecular mechanisms linking IL17A, lipid metabolism, and hepatic fibrosis in order to develop effective treatment approaches.

## Figures and Tables

**Table 1:** Demographic and clinical characteristics of controls and MASLD patients based on the stage of liver fibrosis determined by histology.

	Stage of Liver Fibrosis						
Characteristics	No Fibrosis (F0) (n_1_ = 39)	Mild to Moderate Fibrosis (F1–F2) (n_2_ = 35)	Advanced Fibrosis (F3–F4) (n_3_ = 7)	*p* ^(a)^	Adjusted *p*-Values ^(b)^ for Multiple Comparisons		
					F0 vs. F1–F2	F0 vs. F3–F4	F1–F2 vs. F3–F4
Male sex, *n* (%)	14 (14.44)	14 (12.96)	2 (2.59)	0.8894	-	-	-
Age, years	52.97 (14.43)	55.57 (11.13)	52.86 (4.67)	0.5366	-	-	-
BMI, kg/m^2^	27.46 (5.03)	32.23 (4.49)	32.72 (2.11)	0.00005 *	0.00015 *	0.0004 *	0.8960
Waist circumference, cm	86.90 (14.68)	98.29 (11.42)	99.71 (11.32)	0.0007 *	0.0010 *	0.0508	0.9625
Current smoking, *n* (%)	10 (25.6)	16 (45.7)	2 (28.6)	0.199	-	-	-
SBP, mmHg	130 [118, 145]	130 [125, 145]	140 [135, 153]	0.199	-	-	-
DBP, mmHg	70 [63, 80]	85 [78, 90]	80 [80, 90]	0.001 *	0.0006 *	0.0737	1.000
Liver parameters							
ALT, mg/dL	24 [14, 36]	29 [22, 48]	71 [42, 94]	0.0147 *	0.0817	0.0134 *	0.1966
AST, mg/dL	26 [20, 37]	25 [21, 36]	35 [30, 86]	0.1810	-	-	-
AST/ALT ratio	1.19 [0.83, 1.44]	0.82 [0.70, 1.03]	0.62 [0.55, 1.08]	0.008 *	0.0092 *	0.0471 *	0.8303
Alkaline phosphatase, U/I	177 [1120, 210]	208 [171, 263]	222 [179, 334]	0.007 *	0.0072 *	0.0536	0.9285
GGT, U/I	32 [21, 45]	44 [34, 58]	117 [65, 216]	0.009 *	0.0316 *	0.0152 *	0.3162
Total bilirubin, mg/dL	0.6 [0.5, 0.9]	0.6 [0.5, 0.7]	0.7 [0.6, 0.9]	0.3295	-	-	-
INR	0.99 (0.09)	1.02 (0.11)	1.03 (0.06)	0.408	-	-	-
Frequent comorbidities							
Hypertension, *n* (%)	15 (38.5)	19 (54.3)	6 (85.7)	0.05153	-	-	-
T2DM, *n* (%)	5 (12.8)	10 (28.6)	4 (57.1)	0.02406 *	0.1970	0.0597	0.1970
Obesity, *n* (%)	0 (0.0)	12 (34.3)	4 (57.1)	0.000005 *	0.0001 *	0.0004 *	0.3970
Dyslipidemia, *n* (%)	0 (0.0)	16 (45.7)	3 (42.9)	0.0000005 *	0.000001 *	0.00462 *	1.000
CIC, *n*(%)	1 (2.6)	12 (34.3)	4 (57.1)	0.00005 *	0.00121 *	0.00202 *	0.3970
AP, *n*(%)	2 (5.1)	9 (25.7)	3 (42.9)	0.0063 *	0.0302 *	0.0302 *	0.3870
Statin users, *n* (%)	0 (0.0)	16 (45.7)	2 (28.6)	<0.0001 *	<0.0001 *	0.0406 *	0.6786
Antidiabetic users, *n* (%)	5 (12.8)	10 (28.6)	4 (57.1)	0.02406 *	0.2934	0.0598	0.2934
Antihypertensive users, *n*(%)	15 (38.5)	23 (65.7)	6 (85.7)	0.0121 *	0.0668	0.0729	0.4049

*n* = number of cases; MASLD: metabolically associated steatotic liver disease; SBP: systolic blood pressure; DBP: diastolic blood pressure; ALT: alanine aminotransferase; AST: aspartate aminotransferase; GGT: gamma-glutamyl transferase; T2DM: type 2 diabetes mellitus; CIC: chronic cardiac insufficiency. AP: angina pectoris. Data are expressed as mean (standard deviation) or median [Q1, Q3], where Q1 = first quartile, Q3 = third quartile, or *n* (relative frequency, %). ^(a)^ Estimated from Welch’s ANOVA, Kruskal–Wallis test, and Fisher’s exact test. ^(b)^ Estimated using post hoc tests appropriate for each data distribution (Tukey’s HSD, Games–Howell test, Dunn’s test, or pairwise Fisher’s exact test). * Significant result: *p* < 0.05.

**Table 2:** Distribution of metabolic and genetic factors among patients with advanced, mild to moderate, and no liver fibrosis.

	Stage of Liver Fibrosis						
Characteristics	No Fibrosis (F0) (n_1_ = 39)	Mild to Moderate Fibrosis (F1–F2) (n_2_ = 35)	Advanced Fibrosis (F3–F4) (n_3_ = 7)	*p* ^(a)^	Adjusted *p*-Values ^(b)^ for Multiple Comparisons		
					F0 vs. F1–F2	F0 vs. F3–F4	F1–F2 vs. F3–F4
Metabolic characteristics							
Fasting blood glucose, mg/dL	87 [79, 97]	98 [86, 123]	128 [101, 149]	0.006 *	0.0350 *	0.0076 *	0.1989
Total cholesterol (mg/dL)	176.59 (44.31)	192.51 (34.28)	227.14 (30.01)	0.0065 *	0.1955	0.0067 *	0.0898
HDL cholesterol (mg/dL)	46.97 (12.01)	37.88 (14.04)	32.40 (6.71)	0.0004 *	0.0024 *	0.0065 *	0.4721
LDL cholesterol (mg/dL)	108.08 (46.19)	119.77 (34.64)	145.54 (56.61)	0.0886	-	-	-
Triglycerides (mg/dL)	101 [63, 144]	172 [112, 213]	160 [113, 292]	0.0009 *	0.0009 *	0.0269 *	1.0000
Gene polymorphisms (SNPs)							
*IL17F-A7448G* Genotype AA/AG/GG, *n* (%)	36/2/1 (92.3/5.1/2.6)	27/7/1 (77.1/20.0/2.9)	6/1/0 (85.7/14.3/0.0)	0.2394	-	-	-
Allele A/G, *n* (%)	74/4 (94.9/5.1)	61/9 (87.1/12.9)	10/8 (71.4/5.7)	0.0002 *	0.1450	0.0003 *	0.0110 *
*IL17A-G197A* Genotype GG/GA/AA, *n* (%)	34/3/2 (87.2/7.7/5.1)	17/14/4 (48.6/40.0/11.4)	3/3/1 (42.9/42.9/14.3)	0.0013 *	0.0014 *	0.0423 *	1.000
Allele G/A, *n* (%)	71/7 (91.0/9.0)	48/22 (68.6/31.4)	9/5 (64.3/35.7)	0.0008 *	0.0023 *	0.0338 *	0.7612
*TLR4-Asp299Gly* Genotype Asp/Asp/Asp/Gly/Gly/Gly, *n* (%)	32/7/0 (82.1/17.9/0.0)	20/12/3 (57.1/34.3/8.6)	6/1/0 (85.7/14.3/0.0)	0.0910	-	-	-
Allele Asp/Gly, *n* (%)	71/7 (91.0/9.0)	52/18 (74.3/25.7)	13/1 (92.9/7.1)	0.0163 *	0.0246 *	1.000	0.3479
*TLR4-Thr399Ile* Genotype Thr/Thr/Thr/Ile/Ile/Ile, *n* (%)	37/1/1 (94.9/2.6/2.6)	26/6/3 (74.3/17.1/8.6)	6/1/0 (85.7/14.3/0.0)	0.0943	-	-	-
Allele Thr/Ile, *n* (%)	75/3 (96.2/3.8)	58/12 (82.9/17.1)	13/1 (92.9/7.1)	0.0228 *	0.0360 *	0.9790	0.9790

^(a)^ estimated from Welch’s ANOVA analysis, ANOVA analysis, Kruskal–Wallis test, Fisher’s exact test; ^(b)^ estimated using post-hoc tests appropriated for each data distribution (Tukey’s HSD, Games–Howell test, Dunn’s test, or pairwise Fisher’s exact test); * significant result: *p* < 0.05.

**Table 3:** Metabolic and genetic risk factors for higher liver fibrosis stage in univariable and multivariable ordinal logistic models.

Factors	Liver Fibrosis Stages			
	Univariable Ordinal Regression Model ^(a)^		Multivariable Ordinal Regression Model ^(b)^	
	OR (95% CI)	*p*	Adjusted OR (95% CI)	Adjusted *p*
Demographic characteristics				
Age, years	1.01 [0.98, 1.05]	0.5476	-	-
Sex, male	1.02 [0.43, 2.42]	0.9651	-	-
Anthropometric characteristics				
BMI, kg/m^2^	1.21 [1.10, 1.34]	0.0001 *	1.10 [0.96, 1.27]	0.1710
Waist circumference, cm	1.07 [1.03, 1.11]	0.0005 *	1.03 [0.98, 1.09]	0.2002
Lifestyle-related characteristics				
Current smoking, *n* (%)	1.80 [0.75, 4.39]	0.1885	-	-
Metabolic characteristics				
Fasting blood glucose, mg/dL	1.01 [1.002, 1.03]	0.0237 *	1.001 [0.98, 1.01]	0.9349
Total cholesterol (mg/dL)	1.02 [1.01, 1.03]	0.0045 *	-	-
HDL cholesterol (mg/dL)	0.93 [0.89, 0.96]	0.0003 *	0.96 [0.91, 1.01]	0.1098
LDL cholesterol (mg/dL)	1.01 [1.001, 1.02]	0.0384 *	1.001 [0.99, 1.02]	0.9035
Triglycerides (mg/dL)	1.01 [1.005, 1.02]	0.0004 *	1.003 [1.00, 1.01]	0.3980
Gene polymorphisms (SNPs)				
*IL17F-A7448G* (AG + GG vs. AA genotype)	2.36 [0.75, 7.68]	0.1441	-	-
*IL17A-G197A* (GA + AA vs. GG genotype)	5.93 [2.30, 16.64]	0.0004 *	3.91 [1.33, 12.34]	0.0155 *
*TLR4-Asp299Gly* (Asp/Gly + Gly/Gly vs. Asp/Asp genotype)	1.99 [0.80, 5.04]	0.1392	-	-
*TLR4-Thr399Ile* (Thr/Ile + Ile/Ile vs. Thr/Thr genotype)	3.17 [1.02, 10.48]	0.0504	2.11 [0.51, 8.71]	0.2993

Note. In the ordinal logistic regression, the dependent variable was liver fibrosis stages defined as an ordinal qualitative variable. The odds ratio (OR) quantified the risk for at least a one-stage increase in liver fibrosis severity, but there was no assumption that a one-stage increase had an equal magnitude between liver fibrosis categories; 95% CI: 95% confidence interval of OR. ^(a)^ Studied factors were tested independently in the univariable ordinal logistic regression analysis. ^(b)^ Explicative variables with a *p* < 0.10 in the univariable ordinal logistic regression model were included in the multivariable ordinal logistic model; * significant result: *p* < 0.05; BMI: body mass index, HDL: high-density lipoprotein; LDL: low-density lipoprotein.

**Table 4:** Associations between plasma interleukine levels and studied SNPs in patients with different stages of liver fibrosis.

Groups	SNPs Genotypes Under Dominant Model	IL17A (pg/mL)		IL17F (pg/mL)	
	*IL17F*-*A7448G* Genotypes	Median [Q1, Q3]	*p*	Median [Q1, Q3]	*p*
No Fibrosis (F0) (n_1_ = 39)	AA (*n* = 36)	67.77 [40.49, 98.07]	0.0219 *	7.05 [5.29, 10.02]	0.510
	AG + GG (*n* = 3)	399.46 [256.33, 401.55]		19.91 [12.22, 20.53]	
Mild to Moderate Fibrosis (F1–F2) (n_2_ = 35)	AA (*n* = 27)	146.97 [73.62, 222.56]	0.7532	18.01 [7.31, 26.61]	0.7532
	AG + GG (*n* = 8)	104.60 [94.78, 110.38]		9.82 [7.59, 23.04]	
Advanced Fibrosis (F3–F4) (n_3_ = 7)	AA (*n* = 6)	121.07 [95.87, 171.82]	-	21.75 [13.26, 33.32]	-
	AG + GG (*n* = 1)	NA		NA	
	*IL17A*-*G197A* Genotypes				
No Fibrosis (F0) (n_1_ = 39)	GG (*n* = 34)	76.62 [41.83, 109.74]	0.8997	7.23 [5.24, 12.89]	0.8831
	GA + AA (*n* = 5)	64.94 [45.02, 161.18]		6.85 [5.75, 8.54]	
Mild to Moderate Fibrosis (F1–F2) (n_2_ = 35)	GG (*n* = 17)	168.69 [58.18, 241.45]	0.5859	8.37 [6.25, 26.02]	0.1022
	GA + AA (*n* = 18)	105.03 [89.68, 168.27]		20.81 [8.39, 28.66]	
Advanced Fibrosis (F3–F4) (n_3_ = 7)	GG (*n* = 3)	143.62 [133.10, 162.42]	0.400	8.25 [7.12, 16.24]	0.2286
	GA + AA (*n* = 4)	96.75 [93.67, 129.95]		27.81 [17.27, 36.90]	
		TLR4 (pg/mL)			
		Median [Q1, Q3]	*p*		
	*TLR4*-*Asp299Gly* Genotypes				
No Fibrosis (F0) (n_1_ = 39)	Asp/Asp (*n* = 32)	1316.39 [989.10, 1475.10]	0.3897		
	Asp/Gly + Gly/Gly (*n* = 7)	1217.84 [1035.27, 1275.93]			
Mild to Moderate Fibrosis (F1–F2) (n_2_ = 35)	Asp/Asp (*n* = 20)	1894.18 [1680.49, 1978.21]	0.8155		
	Asp/Gly + Gly/Gly (*n* = 15)	1825.72 [1506.22, 2061.20]			
Advanced Fibrosis (F3–F4) (n_3_ = 7)	Asp/Asp (*n* = 6)	1559.12 [1420.64, 1747.40]	-		
	Asp/Gly + Gly/Gly (*n* = 1)	NA			
	*TLR4*-*Thr399Ile* Genotypes				
No Fibrosis (F0) (n_1_ = 39)	Thr/Thr (*n* = 27)	1259.33 [962.65, 1448.13]	0.3236		
	Thr/Ile + Ile/Ile (*n* = 2)	1426.34 [1398.85, 1453.83]			
Mild to Moderate Fibrosis (F1–F2) (n_2_ = 35)	Thr/Thr (*n* = 26)	1826.76 [1534.23, 1992.73]	0.6103		
	Thr/Ile + Ile/Ile (*n* = 9)	1908.71 [1815.35, 2024.89]			
Advanced Fibrosis (F3–F4) (n_3_ = 7)	Thr/Thr (*n* = 6)	1592.32 [1441.90, 1747.40]	-		
	Thr/Ile + Ile/Ile (*n* = 1)	NA			

*n* = number of cases; median [Q1, Q3] where Q1 = first quartile; Q3 = third quartile; * *p* < 0.05: significant result; NA = not applicable.

## Data Availability

The raw data used in this study can be obtained upon reasonable request from Lucia Maria Procopciuc (lprocopciuc@umfcluj.ro) and Sorina Cezara Coste (secara.sorina@umfcluj.ro; secara.sorina@yahoo.com).
